# Integrative analyses identify HIF-1α as a potential protective role with immune cell infiltration in adamantinomatous craniopharyngioma

**DOI:** 10.3389/fimmu.2022.949509

**Published:** 2022-08-24

**Authors:** Qiang Gao, Jing Luo, Jingjing Pan, Longxiao Zhang, Dengpan Song, Mingchu Zhang, Dingkang Xu, Fuyou Guo

**Affiliations:** ^1^ School of Medicine, Tsinghua University, Beijing, China; ^2^ Department of Neurosurgery, Beijing Tsinghua Changgung Hospital, Beijing, China; ^3^ Department of Rheumatology, Beijing Tsinghua Changgung Hospital, Beijing, China; ^4^ Department of Laboratory Medicine, The First Affiliated Hospital of Wenzhou Medical University, Wenzhou, China; ^5^ Department of Neurosurgery, The First Affiliated Hospital of Zhengzhou University, Zhengzhou, China; ^6^ Department of Neurosurgery, Beijing Hospital, National Center of Gerontology, Institute of Geriatric Medicine, Chinese Academy of Medical Sciences, Beijing, China; ^7^ Graduate School of Peking Union Medical College, Beijing, China

**Keywords:** adamantinomatous craniopharyngioma, weighted gene coexpression network analysis, HIF-1α, immune cell infiltration, immunofluorescence staining

## Abstract

Craniopharyngiomas (CPs) are histologically benign tumors located in the sellar–suprasellar region. Although the transcriptome development in recent years have deepened our knowledge to the tumorigenesis process of adamantinomatous craniopharyngioma (ACP), the peritumoral immune infiltration of tumor is still not well understood. In this study, weighted gene coexpression network analysis (WGCNA) was applied to identify different gene modules based on clinical characteristics and gene expression, and then, the protein–protein interaction (PPI) network with the Cytohubba plug-in were performed to screen pivotal genes. In addition, immune cell infiltration (ICI) analysis was used to evaluate the immune microenvironment of ACP patients. In total, 8,568 differential expression genes were identified based on our datasets and two microarray profiles from the public database. The functional enrichment analysis revealed that upregulated genes were mainly enriched in immune-related pathways while downregulated genes were shown in the hormone and transduction of signaling pathways. The WGCNA investigated the most relevant modules, and 1,858 hub genes was detected, from which the PPI network identified 14 pivotal genes, and the Hypoxia-inducible factor 1-alpha (HIF-1α) pathway including four critical genes may be involved in the development of ACP. Moreover, naïve CD4+ and CD8+ T cells were decreased while specific subtypes of T cells were significantly increased in ACP patients according to ICI analysis. Validation by immunofluorescence staining revealed a higher expression of HIF-1α in ACP (ACP vs. control) and adult-subtype (adult vs. children), suggesting a possible state of immune system activation. Notably, children with low HIF-1α scores were related to the hypothalamus involvement and hydrocephalus symptoms. In this study, we successfully identified HIF-1α as a key role in the tumorigenesis and development of ACP through comprehensive integrated analyses and systematically investigated the potential relationship with immune cells in ACP. The results may provide valuable resources for understanding the underlying mechanisms of ACP and strengthen HIF-1α as a potential immunotherapeutic target in clinical application.

## Introduction

Craniopharyngiomas (CPs) are common benign intracranial tumors accounting for 0.5–2 new cases per 1 million population (1). Radical resection has been considered the preferred treatment, and postoperative adjuvant radiotherapy can also prolong the progression-free survival (PFS) for patients with subtotal resection (2, 3). Overall, due to the anatomical location adjacent to vital brain structures, such as the optic chiasm and hypothalamus, surgical resection often leads to severe endocrine deficits and neuropsychological disorders in the long-term follow-up. The two types of CPs, adamantinomatous craniopharyngiomas (ACPs) and papillary craniopharyngiomas (PCPs), vary from onset ages, pathological characteristics, and genetic backgrounds. Although ACPs have been considered as WHO I grade tumors, they are characterized by malignant biological behaviors: finger-like protrusion to brain tissues (4), which might explain the high recurrence rate (approximately 10.3% within 5 years) for patients receiving total resection surgeries (5). However, the underlying mechanism of ACPs was still unclear and there was also still a lack of novel index to improve the treatments of ACP patients.

Recently, high-throughput sequencing technologies have brought us a new understanding of the genetic profiles and pathogenesis of ACPs. Apps et al. revealed MAPK/ERK pathway overexpression in ACPs, suggesting that the MEK inhibitor, trametinib, might serve as a novel therapeutic target (6). Meanwhile, the IL-6 inhibitor, tocilizumab, was also proven to be effective in some specific cases (7). In addition, our previous studies also investigated essential transcript factor (TF)–lncRNA pairs based on an integrated algorithm, which provided new insights into the underlying mechanism of ACP development (8). Currently, the accumulation of beta-catenin caused by CTNNB1 mutations has been recognized as the driver mutations in ACPs (9–11). Studies focusing on the murine ACP model raised a new hypothesis in initiating tumor formation: tumors derived from SOX2− cells transformed by SOX2+ pituitary stem cells expressing beta-catenin in the senescence-associated secretory phenotype (SASP) (12). However, the conception that stem cells promoted tumor development in a paracrine manner was not consistent with the traditional model in which stem cells become tumor-initiating cells. Thus, further exploration to the undiscovered mechanism of ACP pathogenesis and immune microenvironment is urgently needed in the future.

In this research, we systematically explored the immune profile of ACPs in the GEO datasets and further verified the corresponding results through our own sequenced datasets and immunofluorescence staining experiments. This study first revealed the potential role of HIF-1α in the development of ACPs and intrinsic correlation with immune cell infiltrations (ICIs). In conclusion, our group may provide a new insight to tumorigenesis mechanism and potential therapeutic target for ACP patients.

## Methods

### Patient preparation and data collection

A total of 12 ACP patients and five health control (HC) cohorts with their brain tissues were recruited from the First Affiliated Hospital of Zhengzhou University to conduct RNA sequencing from 1 June 2020 to 30 December 2020. Tumor samples were obtained after the surgical treatment, and the purity of ACP samples was further identified through hematoxylin and eosin staining and β-catenin immunostaining on histological sections. In addition, normal brain tissues from five patients with craniocerebral trauma were used for comparative purposes and all tissue samples were conserved in RNAlater^®^ within -80°C for subsequent RNA sequencing. Corresponding clinical features and complete ACP-related tumor characteristics were also collected including age, gender, clinical symptom, tumor size, location, and prognosis status. This study was approved by the Ethics Committee of the First Affiliated Hospital of Zhengzhou University, and informed consent was written by all participants for their enrollments.

To ensure the reliability and accuracy of our results, we also downloaded two eligible microarray datasets from the GEO database (https://www.ncbi.nlm.nih.gov/geo/) with the following inclusion criteria: 1) including the gene expression data of ACP patients and HC cohorts while excluding papillary craniopharyngioma or other intracranial tumors; 2) using brain tissue samples for subsequent sequencing analysis rather than blood samples; and 3) the sequenced platform must include more than 5,000 genes, and different datasets need to be from the same platform to avoid unnecessary deletion.

### RNA sequencing and quality control

Total RNA from frozen brain tissues were isolated using a TRIzol^®^ Reagent (Invitrogen) and the Nano Photometer^®^ spectrophotometer (IMPLEN, CA, United States) was applied to check the RNA purity. Using the Agilent RNA 6000 Nano kit (Agilent Technologies, CA, United States), we estimated the RNA integrity numbers (RIN) of total RNA and only kept high-quality RNA with RIN >7 to further construct the cDNA library. Through using the NEBNext Ultra II RNA Library Kit (NEB, Ipswich, United States) with 3-μg RNA per sample, sequencing libraries were constructed and sequenced on the Illumina HiSeq platform. Subsequently, we applied Cutadapt adapters to trim the raw reads and used the FastQC software to obtain the quality control reports of sequence reads. Finally, the sequencing data were aligned to the human reference genome (hg38) using the STAR software and the count data were normalized as the fragments per kilobase of transcript per million (FPKM) data after filtering read count files with low expression.

Two eligible microarray profiles were downloaded from the GEO database with the FPKM format, and the “impute” package (13) was used to impute few missing values based on the k-nearest neighboring (KNN) imputation algorithm. Then, gene probes with low expression levels were also eliminated and the “ComBat” algorithm in the “sva” package was further applied to remove the batch effects from different microarray data (14). Finally, the normalization process was performed using the “normalizeWithinArrays” and “normalizeBetweenArrays” functions of the “limma”package and the probe labels were transferred into corresponding gene symbols based on the platform’s annotation file (15).

### Identification of differentially expressed genes for adamantinomatous craniopharyngiomas

The transcriptomic difference between ACP and HC cohorts was exhibited by a two-dimensional principal component analysis (PCA) using the “princomp” function, and the expression of the top DEGs was displayed in heatmaps using the “pheatmap” package. The DEGs was screened by the “limma” package based on the following criteria: the absolute value of log2 fold change (FC) >1 and adjusted p-value<0.05. Subsequently, all DEGs were visualized in volcano plots using the “ggplot2” package (16) and Kyoto Encyclopedia of Genes and Genomes (KEGG) pathway enrichment analyses were conducted by the “clusterProfiler” package (17).

### Weighted gene coexpression network analysis

To investigate the correlation between clinical characteristics and gene expression, the top 75% genes with the highest variance in ANOVA analysis were extracted to construct the coexpression network using the WGCNA package (18). Then, the adjacency matrix was further transformed into a topological overlap matrix (TOM) and hierarchical clustering through TOM was applied to identify different gene modules. During this process, the soft threshold power was chosen as 16 when 0.9 was set as the scale-free R^2^ threshold, and the least genes in each module were set as 30. Moreover, the similar modules with more than 75% cut height in clustering trees were merged into same modules and hub genes were identified with a gene significance (GS) value >0.6 and module membership (MM) value >0.9 (19).

### Identification of hub genes based on protein–protein interaction networks

Common hub genes were ultimately identified based on the combination of previously found DEGs and significant modules *via* WGCNA, and the KEGG enrichment analysis was further performed by the ClueGO plug-in (20). Subsequently, the protein–protein interaction (PPI) networks of the above hub genes were obtained from the STRING database and structured using the Cytoscape soft (21). To further identify pivotal genes in the network, the Cytohubba plug-in was applied to screen the top 20 genes with 12 algorithms (22), of which genes discriminated by more than six algorithms were identified as essential potential participators in the mechanism of ACPs (23).

### Immune cell infiltration and correlation analysis

To further evaluate the ICI characteristics of brain tissues in ACP patients, the Immune Cell Abundance Identifier (ImmuCellAI) online tool was applied to transform the sequencing profiles into immune infiltration scores based on the abundance of 24 immune cell types (24). The proportion of significant immune cells was exhibited in scale diagrams using the “ggplot2” package, and the correction between the expression of vital genes and the infiltration scores of multiple immune cells were also performed *via* “Spearman” methods. In addition, the comparison of various immune cells between ACP and HC subgroups with distinct degrees of immune infiltration scores was performed by the Wilcoxon test.

### Clinical correlation analysis and immunofluorescence validation

To increase the reliability and accuracy of diagnosis for our own ACP patients, we also collected and displayed their corresponding radiographic and pathological characteristics. Moreover, we further divided the ACP patients into two groups with high- or low-expression levels of HIF1α based on a compositive parameter, candidate scores, which was used to represent standardized gene expression and was also calculated in previous studies (25). Firstly, we calculated the mean values and standard deviation (SD) of HIF1α in HC groups to normalize its expression for each ACP patient. Subsequently, the standardized HIF-1α levels of ACP patients were recalculated based on the following calculation formula:


$$\text{Candidate scores i }\left(HIF1\alpha \;\right)=\frac{\text{HIF}1\text{α i }\left(\text{ACP}\right)-\text{Mean }\left(\text{HC}\right)}{\text{SD }\left(\text{HC}\right)}$$

where “HIF-1α i (ACP)” represents the expression of HIF-1α in each ACP patient. Finally, we applied the double normal distribution model to identify the threshold of candidate HIF-1α scores using the “mixtools” package (26) and performed the comparison of clinical characteristics between high- and low-HIF-1α subgroups.

ACP tissues were prepared for double-labeled immunofluorescence. Briefly, the slices were incubated with mouse anti β-catenin (1:1,000, Servicebio, Wuhan, China) and rabbit anti-HIF1α (1:1,000, Servicebio, Wuhan, China). Next, a goat anti-rabbit antibody conjugated to Cy3 (1:300, Servicebio, Wuhan, China) or a goat anti-mouse antibody conjugated to FITC (1:500, Servicebio, Wuhan, China) was added and incubated for 1 h at room temperature. Finally, DAPI was applied to visualize the cell nucleus.

### Statistical analysis

All the statistical analyses were performed in R software version 3.6.1 (https://www.r-project.org/). The Wilcox rank-sum test was used to compare continuous variables, and the chi-square test was applied to compare classified variables. The two-tailed P-value<0.05 was considered statistically significant.

## Results

### Exploring the difference of gene expression between adamantinomatous craniopharyngioma and control groups

**Figure 1:**
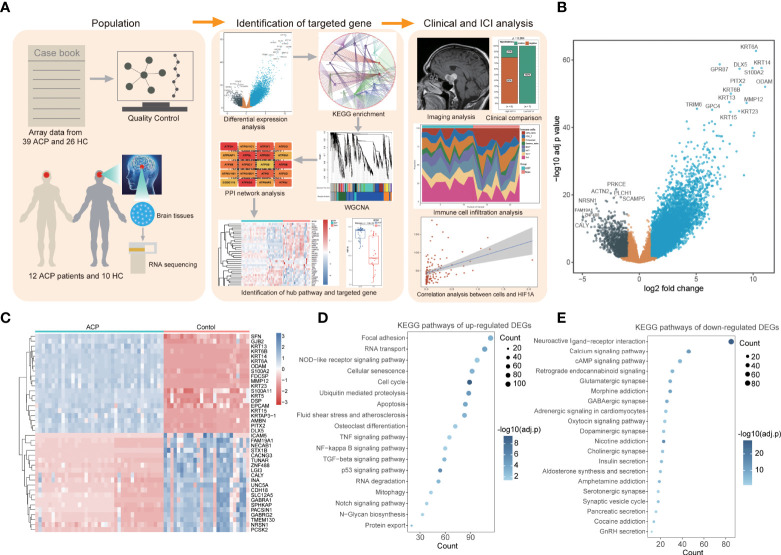
Identification of differentially expressed genes (DEGs) and functional enrichment in adamantinomatous craniopharyngiomas (ACPs). **(A)**. The workflow of the whole process in this study. **(B)**. The volcano map displayed a total of 8,568 DEGs including 7,247 upregulated and 1,321 downregulated genes. **(C)**. The heatmap of top 20 DEGs exhibited amazing discriminative capacity between ACP patients and control cohorts. **(D, E)**. The KEGG enrichment analysis revealed that most upregulated DEGs were enriched in the cell biological process and immune-activated signaling pathways. **(D)** while downregulated signatures were majorly enriched in the secernent disorder of multiple hormones and signal regulation pathways **(E)**.

The workflow of the whole process in this study is shown in **Figure 1A**. According to the selection criteria, two sole eligible microarray profiles (GSE68015 and GSE94349) were screened to preprocess and merged for subsequent analysis. Notably, prominent batch effects existed between two datasets and it was remarkably reduced after removing batch effects (**Supplementary Figure 1A**). PCA analysis displayed distinct gene expression patterns between the ACP patients and control cohorts and potential inner heterogenicity in ACP groups (**Supplementary Figure 1B**). According to the threshold criteria [adjusted p-value< 0.05 and absolute (log2 FC) > 1], a total of 8,568 DEGs were identified, including 7,247 upregulated and 1,321 downregulated genes, and the top 20 DEGs exhibited amazing discriminative capacity for ACP patients (**Figures 1B, C**). Furthermore, the functional enrichment analysis revealed that most upregulated DEGs were enriched in the cell biological process and immune-activated signaling pathways while downregulated signatures were majorly enriched in the secernent disorder of multiple hormones and signal regulation pathways (**Figures 1D, E**).

### Identification of adamantinomatous craniopharyngioma-related gene modules *via* weighted gene coexpression network analysis

**Figure 2:**
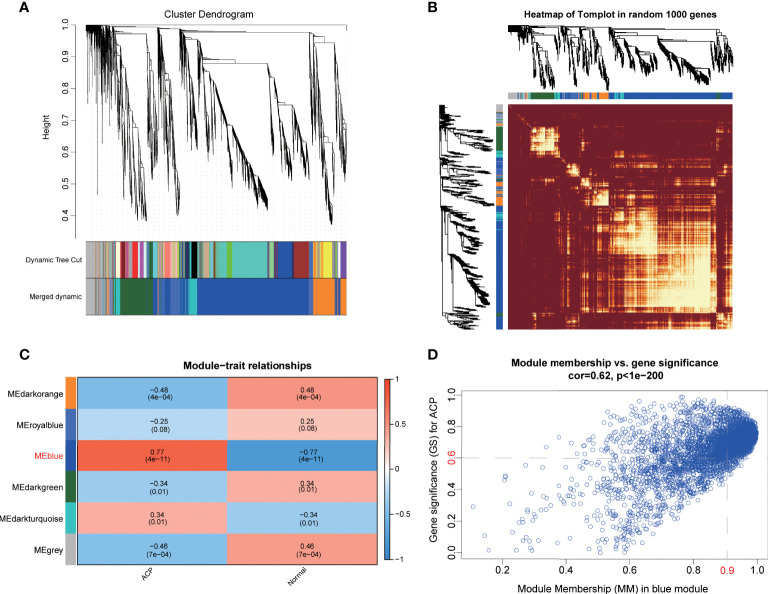
Screening pivotal modules and hub genes closely associated with ACPs by weighted gene coexpression network analysis (WGCNA). **(A)**. The dendrogram of all genes showing that a total of six modules were eventually identified after merging similar modules. **(B)**. The heatmap displayed the interrelation and stability of these modules based on the expression of random 1,000 genes. **(C)**. The blue module showed the highest correlation with ACP patients, with the coefficient = 0.77 and P= 4e-11. **(D)**. Scatter plot of the blue module identified 1,858 hub genes to manifest the module’s characteristics based on the screening criterion with high module membership (MM) and gene significance (GS) values.

To further find out the key gene modules most associated with ACP patients, we performed the WGCNA analysis based on the public gene expression profiles. Through setting the soft-thresholding power as 16 with corresponding scale-free R^2^ as 0.9, and the cut height as 0.25, a total of six modules were eventually identified in this study, of which the gray module included non-clustering genes (**Figure 2A**; **Supplementary Figure 1C**). Furthermore, the heatmap based on the expression of random 1,000 genes also displayed the interrelation and stability of these modules (**Figure 2B**) and the blue module was considered as the most highly correlated with ACP patients (coefficient = 0.77, P= 4e-11) (**Figure 2C**). To further explore the correlation between GS values for the disease and MM values in the blue module, fitting correlation analysis manifested that the blue module contained a total of 4,069 genes (correlation coefficient = 0.62, p = 1e–200). Moreover, 1,858 hub genes were further chosen to manifest the module’s characteristics based on the screening criterion with high MM and GS values (**Figure 2D**).

### Defining HIF-1α as a critical factor in adamantinomatous craniopharyngioma patients

**Figure 3:**
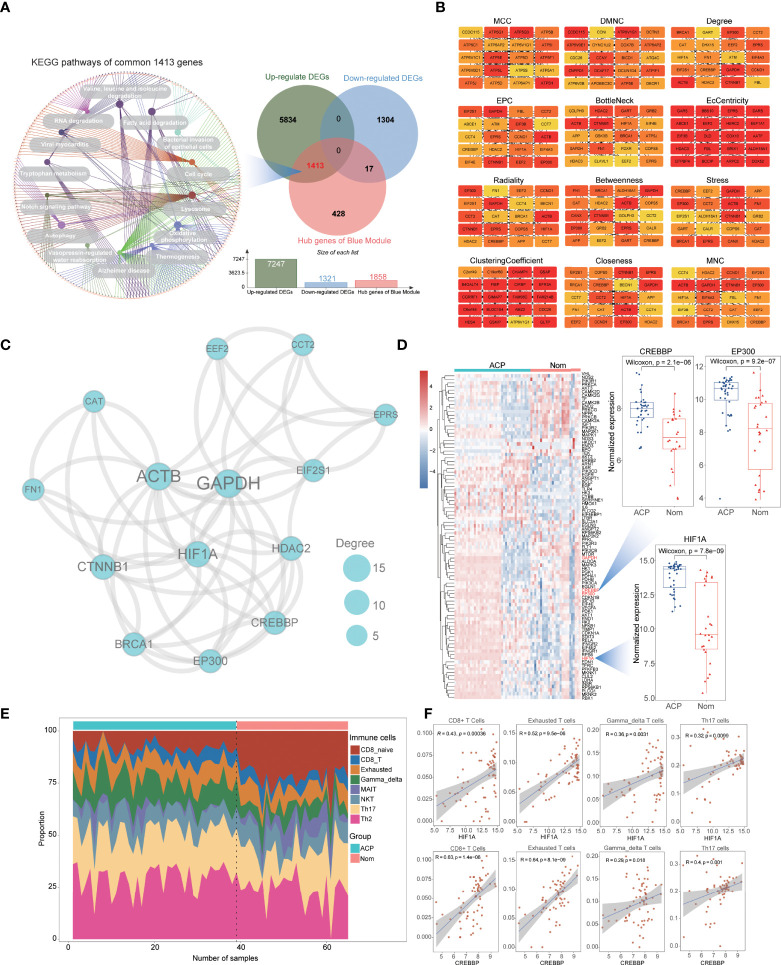
Identification of vital genes and immune cell infiltration (ICI) analysis for ACPs. **(A)**. Combined with previous DEGs and blue-module genes, the Venn plot showing common 1,413 upregulated hub genes and KEGG enrichment analysis indicated that they were significantly enriched in biological processes associated with energy metabolism regulation including fatty acid degradation, tryptophan metabolism, the Notch signaling pathway, autophagy, and oxidative phosphorylation. **(B)**. The top 20 genes from the PPI network with 12 algorithms using the Cytohubba plug-in. **(C)**. The protein–protein interaction (PPI) network of the HIF-1α signaling pathway using hub genes. The nodes represent the degree value. **(D)**. The heatmap showing the different expression of genes from all the HIF-1α signaling pathways between ACP and control cohorts and three essential genes (HIF-1α, CREBBP, and EP300) were significantly increased in ACP patients. **(E)**. ICI analysis revealed that naïve CD4+ and CD8+ T cells were significantly decreased while specific subtypes of T cells were significantly activated in ACP patients including gamma–delta T cells, NK T cells, and Th17 and Th2 cells. **(F)**. Correlational analysis displayed a significantly positive association between specific T cells and HIF-1α or CREBBP.

Combined with previous DEGs, we noticed that most blue-module genes were consisted with upregulated DEGs (1,413 hub genes) and KEGG enrichment analysis indicated that these common hub genes were significantly enriched in biological processes associated with energy metabolism regulation including fatty acid degradation, tryptophan metabolism, the Notch signaling pathway, autophagy, and oxidative phosphorylation (**Figure 3A**). Through constructing the PPI networks of 1,413 hub genes, we successfully identified 14 critical genes (*ACTB, BRCA1, CAT, CCT2, CREBBP, CTNNB1, EEF2, EIF2S1, EP300, EPRS, FN1, GAPDH, HDAC2*, and *HIF-1α*) with ≥6 approval of algorithms in the Cytohubba plug-in (**Figure 3B**; **Table 1**). Interestingly, these critical genes were also enriched in the major pathways of 1,413 hub genes and the HIF-1α signaling pathway might participate in the potential mechanism of ACP patients according to literature searching including four critical genes (*CREBBP, HIF-1α, EP300*, and *GAPDH*, **Figure 3C**). The heatmap of the HIF-1α signaling pathway also exhibited their discriminative capacity between ACP and control cohorts, and the expression of *CREBBP, HIF-1α*, and *EP300* were all significantly elevated in ACP patients (**Figure 3D**).

**Table 1:** Identification of hub genes through Cytohub plug-in with 12 algorithms.

Gene	Number	Approbatory algorithms							
*ACTB*	8	Betweenness	BottleNeck	Closeness	Degree	EPC	MNC	Radiality	Stress
*BRCA1*	7	Betweenness	BottleNeck	Closeness	Degree	MNC	Radiality	Stress	
*CAT*	6	Closeness	Degree	MNC	Radiality	Stress			
*CCT2*	7	Closeness	Degree	EPC	MNC	Radiality	Stress		
*CREBBP*	7	Closeness	Degree	EPC	MNC	Radiality	Stress		
*CTNNB1*	8	BottleNeck	Closeness	Degree	EPC	MNC	Radiality	Stress	
*EEF2*	9	BottleNeck	Closeness	Degree	EcCentricity	EPC	MNC	Radiality	Stress
*EIF2S1*	6	Closeness	Degree	EPC	MNC	Radiality	Stress		
*EP300*	7	Closeness	Degree	EPC	MNC	Radiality	Stress		
*EPRS*	9	BottleNeck	Closeness	Degree	EcCentricity	EPC	MNC	Radiality	Stress
*FN1*	7	BottleNeck	Closeness	Degree	MNC	Radiality	Stress		
*GAPDH*	8	BottleNeck	Closeness	Degree	EPC	MNC	Radiality	Stress	
*HDAC2*	8	BottleNeck	Closeness	Degree	EPC	MNC	Radiality	Stress	
*HIF-1α*	6	Closeness	Degree	EPC	MNC	Radiality			

To expound the characteristics of ICI in APC patients, we performed the ImmuCellAI algorithm to compare the infiltration levels of 24 different immune cells. It revealed that naïve CD4+ and CD8+ T cells were significantly decreased while specific subtypes of T cells were significantly activated in ACP patients including gamma–delta T cells, natural killer NK T cells, and Th17 and Th2 cells (**Figure 3E**; **Supplementary Figure 1D**). Interestingly, correlational analysis displayed a significantly positive association between specific T cells and these critical genes, especially HIF-1α, suggesting the potential connection of HIF-1α and ICI characteristics in ACP patients (**Figure 3F**; **Supplementary Figure 1E**).

### Validation of HIF-1α based on RNA-seq data from clinical adamantinomatous craniopharyngioma patients

**Figure 4:**
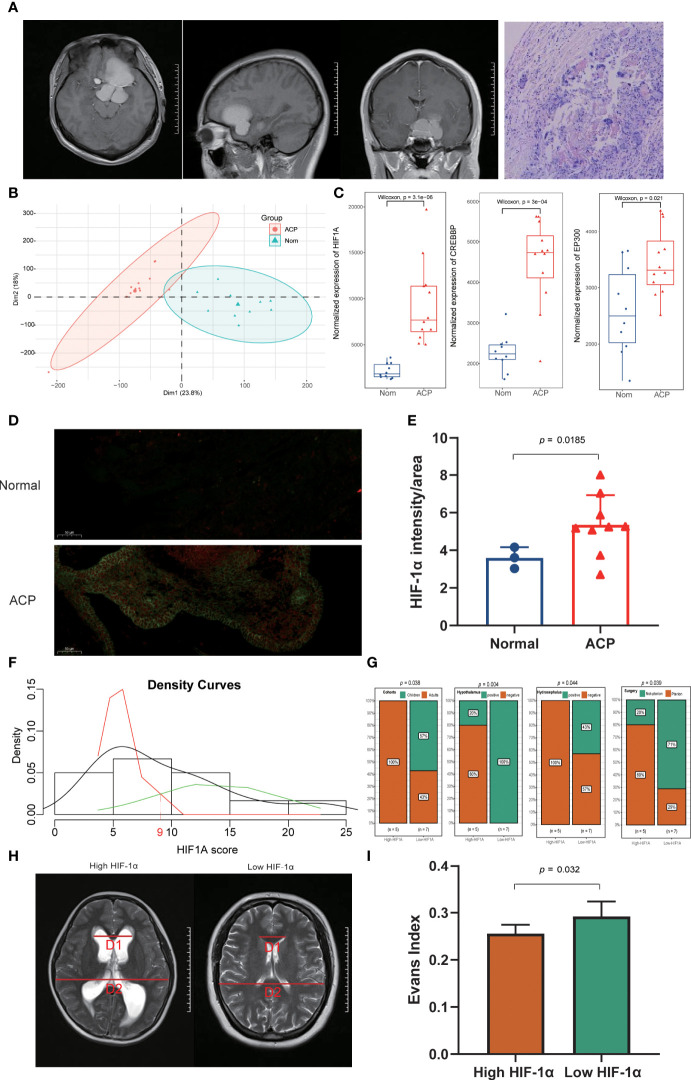
Exploration and validation of clinical characteristics of HIF-1α in ACP patients. **(A)**. ACP patients enrolled in our study displayed classical neuroimaging and pathological abnormality. **(B)**. The principal component analysis (PCA) exhibited the inner homogeneity of ACPs different from the health controls at the gene expressional levels. **(C)**. Higher expression levels of both HIF-1α and CREBBP were detected in ACP patients, while there was no statistical difference for EP300 compared with the expression in control groups. **(D, E)**. The immunofluorescence staining of ACP histological sections consistently validated their higher expression at protein levels in ACP patients. **(F)**. ACP patients were successfully divided into high- and low- HIF-1α score subgroups based on the Candidate score 9 as the expressional threshold via the double normal distribution model. **(G)**. The low-HIF-1α score cohorts included all children patients, exhibited more severe clinical phenotypes including hypothalamus involvement and hydrocephalus, and received non-pterional approaches. **(H, I)**. The low-HIF-1α score patients exhibited more significant cerebroventricular expansion than that of high-HIF-1α patients with a higher Evans index.

To further validate the expression levels and explore the potential clinical characteristics of HIF-1α in ACP, we recruited 12 ACP patients (four children and eight adults) and five normal controls with RNA-seq and comprehensive clinical records (**Table 2**). All these patients displayed classical neuroimaging and pathological abnormality (**Figure 4A**), and the PCA exhibited the inner homogeneity of cohorts different from the health controls at the gene expressional levels (**Figure 4B**). Except for *EP300*, higher expression levels of both *HIF-1α* and *CREBBP* were all detected in ACP patients compared with that in control groups (**Figure 4C**) and the immunofluorescence staining of ACP histological sections consistently validated their higher expression at protein levels in ACP patients (**Figures 4D, E**). These findings accordingly confirmed that HIF-1α was enriched in the disordered brain tissues of ACP patients.

**Table 2:** Clinical characteristics of adamantinomatous craniopharyngioma patients in our sequencing datasets.

Terms	ACP (n=12)	Terms	ACP (n=12)	Terms	ACP (n=12)
Age (years)		Optic nerve		Pathology	
≤18y; n (%)	4 (33.3%)	positive; n (%)	11 (91.7%)	adamantinomatous; n (%)	12 (100%)
>18y; n (%)	8 (66.7%)	negative; n (%)	1 (8.3%)	squamous papillary; n (%)	0 (0%)
Gender		Location		Resection status	
Female; n (%)	4 (33.3%)	suprasellar; n (%)	5 (41.7%)	gross total; n (%)	3 (25%)
Male; n (%)	8 (66.7%)	suprasellar and inside; n (%)	7 (58.3%)	subtotal; n (%)	9 (75%)
Disease time (months; median[Q1–Q3)]	0.5 (0.275-5.25)	Calcification		Radiotherapy	
PFS [months; median(Q1–Q3)]	13 (11.75-18)	positive; n (%)	10 (83.3%)	positive; n (%)	0 (0%)
Tumor size [mm^3^; median(Q1–Q3)]	8844.63 (6320.49-31934.29)	negative; n (%)	2 (16.7%)	negative; n (%)	12 (100%)
Endocrine		Hydrocephalus		Outcome	
Positive; n (%)	10 (83.3%)	positive; n (%)	3 (25%)	alive; n (%)	10 (83.3%)
Negative; n (%)	2 (16.7%)	negative; n (%)	9 (75%)	death; n (%)	2 (16.7%)
Hypothalamus involvement		Surgery approach			
positive; n (%)	8 (66.7%)	by pterion; n (%)	6 (50%)		
negative; n (%)	4 (33.3%)	by non-pterion; n (%)	6 (50%)		

Based on the double normal distribution model, the Candidate score 9 was set as the threshold for HIF-1α scores and these ACP patients were successfully divided into high- and low- HIF-1α score subgroups (**Figure 4F**). Notably, the low-HIF-1α score cohorts included all children patients and exhibited more severe clinical phenotypes including hypothalamus involvement and hydrocephalus (**Figure 4G**; **Supplementary Figure 2B**). In addition, the craniotomy by a pterional approach has been generally acknowledged as the most common and safest surgical procedure for ACP patients but more than half of the low-HIF-1α score patients received non-pterional approaches, implying that more complex and serious lesions might occur in this subtype (**Figure 4G**). We also calculated the Evans index to evaluate the degree of hydrocephalus, and it also revealed that the low-HIF-1α score patients exhibited more significant cerebroventricular expansion than that of high-HIF-1α patients (**Figures 4H, I**).

### Exploring the potential role of HIF-1α in immunoregulation and immunotherapy

**Figure 5:**
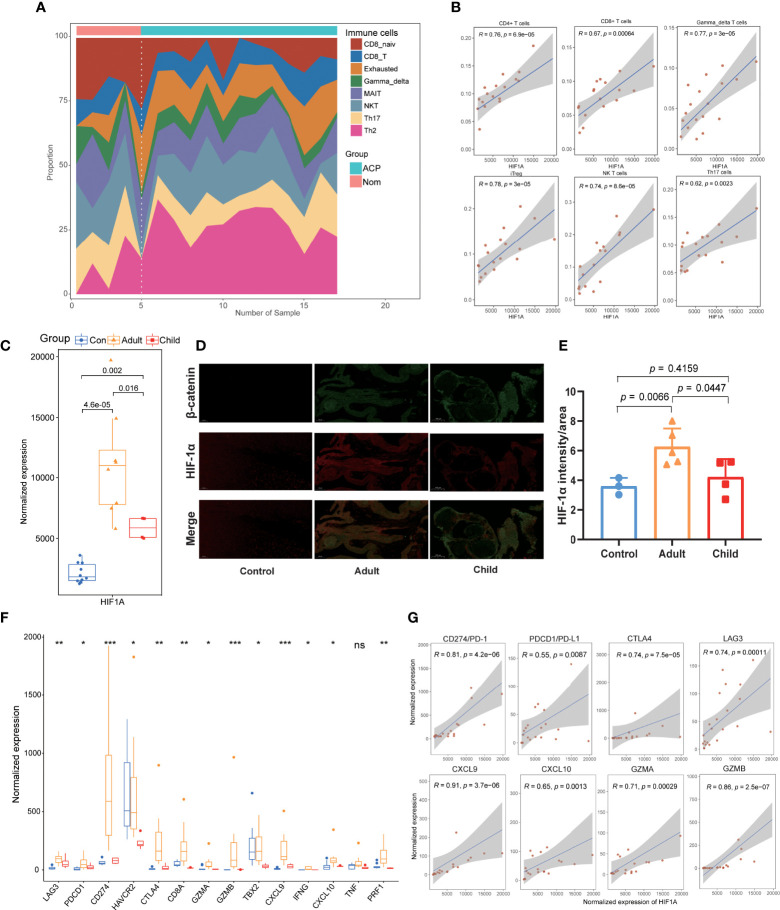
Validation of HIF-1α’s immunological characteristics and the correlation with special age distribution. **(A)**. Our own sequencing data validated the infiltration of multiple immune cells in the tissues of ACP patients including B cells, CD4+ T cells, CD8+ T cells, Th17 cells, NK T cells, gamma–delta T cells, and Th2 cells. **(B)**. The correlation analysis revealed that HIF-1α was significantly positively associated with the infiltration of multiple immune cells. **(C)**. Higher expression levels of HIF-1α were detected in adult-ACP than that of children patients. **(D, E)**. Immunofluorescence staining validated higher levels of HIF-1α in adult-ACP than that of children patients. **(F)**. The expression levels of immune checkpoints and inflammatory factors were significantly upregulated in the adult-ACP patients than child subtypes and controls. **(G)**. HIF-1α exhibited a positive relationship to the expression of immune-associated genes. *p < 0.05; **p < 0.01; ***p < 0.001; ns, no significance.

The immune activation status was also validated in ACP patients based on our sequencing datasets, and multiple immune cells were infiltrated in the tissues of ACP patients including B cells, CD4+ T cells, CD8+ T cells, Th17 cells, NK T cells, gamma–delta T cells and Th2 cells (**Figure 5A**; **Supplementary Figure 2A**). Consistently, the correlation analysis also revealed that HIF-1α was significantly positively associated with the infiltration of multiple immune cells (**Figure 5B**; **Supplementary Figure 2C**). Of interest, we also detected the different expression levels of HIF-1α between children- and adult-ACP patients (**Figure 5C**) and the immunofluorescence staining further validated higher levels of HIF-1α in adult than that of children patients (**Figures 5D,E**). Furthermore, the comparison of immune-associated genes among the controls and child- and adult-ACP patients was performed, including immune checkpoints and inflammatory factors, and most genes were upregulated in the adult patients (**Figure 5F**). Moreover, the expression of these immune-associated genes was positively related to the expression of HIF-1α, consistent with a significant association between HIF-1α and ICI (**Figure 5G**). Overall, these results demonstrated the differential expression levels between child- and adult-ACP patients and supported the immune-activated status in adult patients with higher levels of HIF-1α.

## Discussion

Craniopharyngioma is a common benign tumor in the sella region and often causes severe endocrine disorders to patients with persistent growth in a specific location. Postoperative endocrine deficit and neuropsychological disorder impede the further recovery and reintegration of patients, especially for children patients. Due to the invasive growth of tumors and functional damages from surgical resection, the prognosis of ACP remains relatively poor and there is still the lack of further understanding to the potential pathogenesis of ACP patients. In this study, we firstly identified HIF-1α as a pivotal regulator participating in the pathogenesis of ACP and found that it was significantly associated with clinical phenotypes including hydrocephalus and hypothalamus involvement based on clinical characteristics. Moreover, the expression of HIF-1α exhibited prominent age-specific distribution based on our own transcriptome profiles and immunofluorescence staining, and child-ACP patients exhibited a higher level of HIF-1α expression than the adult cohorts. In addition, the ICI analysis demonstrated that HIF-1α displayed an outstanding positive association with the activation of immune cells and a high expression of immune checkpoints. In short, the comprehensive integrated analysis provides a new pathological insight into the mechanism underlying the development and serve as a potential therapeutic target of ACP.

Previous studies have also indicated that HIF-1α extremely promoted tumor growth and played a key role in the development of multiple tumors. In the patients with glioblastoma, HIF-1α promoted tumor stemness and self-renewal, and inhibited differentiation, which was associated with the disease progression (27). Furthermore, similar fundings were also reported in ovarian cancer and other tumors, implying the malgenic roles of HIF-1α in the progression of tumors (28). In our study, through the multiplex analysis based on the transcriptome profiles of ACP, we firstly found that HIF-1α expression was significantly upregulated in ACP patients, whereas low HIF-1α scores in the ACP group were correlated with severity of ventriculomegaly and the onset age. This is to say, the role of HIF-1α in tumor and non-tumor groups and ACP populations is inconsistent. Moreover, ICI analysis further indicated that HIF-1α was positively correlated with multiple immune cells, especially with CD4+, CD8+, and γΔ T cells, and the corresponding phenomenon was also validated in tumor tissues with high-throughput sequencing and immunofluorescence staining experiments. These results indicated that HIF-1α might participate in both the pathogenesis and antitumor effects with the activation of the adaptive immune response in ACP patients, suggesting that HIF-1α might be a potential immune regulator in ACP.

HIF-1α, an essential transcription factor involved in regulating the aggressive phenotype of tumors under hypoxic conditions, appears to be a two-edged sword for the prognosis of patients with different tumors. In malignant tumors, overexpressed HIF-1α connived tumor cells to adapt to hypoxic environments, maintain nutrient acquisition, and promote further tumor growth. Noteworthy, some studies also found the protective role of HIF-1α in the invasion of tumors. For example, Ashutosh et al. demonstrated that the loss of HIF-1α could promote the invasion and metastasis of pancreatic ductal adenocarcinomas (PDACs) *via* increasing PPP1R1B expression and degrading the p53 in mice models (29). However, for benign neoplasms, especially intracranial benign tumors, little attention has been paid to HIF-1α. According to the DEGs and tissue immunofluorescence validation in our study, the expression of HIF-1α was significantly increased in the ACP tissue, consistent with the findings of a previous study (30), and HIF-1α seems to play a potential novel role in the biological process of the craniopharyngioma.

Based on deeper analysis and integrative scoring, we further explored the clinical characteristics between the high- and low-HIF-1α expression subgroups. Although the occurrence of cerebral hypoxia contributed to the development of chronic adult hydrocephalus (31), patients with a high expression of HIF-1α were all adults and no hydrocephalus occurred in this cohort. To explore the relationship between ventricular dilatation and HIF-1α expression, we assessed the imaging features of patients in both groups using the Evans index. As a reliable index, the Evans index has been widely used for the evaluation of normal senile brain atrophy and pathological hydrocephalus (32). Interestingly, it revealed that the degree of ventriculomegaly was more severe in the low-HIF-1α groups. Therefore, the obstructive hydrocephalus caused by CP may be related to the lack of HIF-1α promoting tumor growth, like the tumor suppressor effect of high HIF-1α expression in renal cancer (33). Meanwhile, Persson C. et al. reported that HIF-1α was negatively correlated with the presence of blood vessels (34), so the low expression of HIF-1α might lead to the proliferation of blood vessels in ACP. We speculate that in the absence of HIF-1α inhibition, the cyst wall of ACP rapidly expands and the cyst fluid fills quickly, resulting in the gradual increase of space occupancy, blocking the circulation of cerebrospinal fluid, and causing ventricular dilatation over time and hydrocephalus. Overall, a high expression of HIF-1α may play a role in promoting tumor growth, but in the pediatric population, it is negatively correlated with severe clinical features. This also leads us to a fundamental difference in the development of child-type and adult-type ACP. We therefore cautiously believe that HIF-1A differs between the two populations (ACP vs. control, children vs. adults) and needs to be validated in a larger cohort.

It has been well known that ACPs show a bimodal incidence, peaking in both childhood and adults at 45–60 years; however, the underlying etiology of this special age-related distribution is still unclear. Therefore, we divided our sequenced patients into adult and children groups to further explore their potential correlation between HIF-1α and clinical characteristics. Although the size of samples was limited, the PCA indicated excellent inner homogeneity and the sequencing results also demonstrated higher expression levels of HIF-1α in adults than that of children patients, which was further validated *via* tissue immunofluorescence staining. Children are in a critical period of growth and development, and the nervous system consumes a larger amount of oxygen than that in adults (35); therefore, children cohorts possess more intracranial oxygen supply and lower HIF-1α levels. HIF-1α was ubiquitously expressed in hypoxic tissues, and it was worth noting that the tissues of most healthy adults were not hypoxic, so HIF families were usually present in low amounts. Different isoforms of HIF transcription factors played different roles, with HIF-1α rapidly activated during acute and severe hypoxia, while HIF-2a accumulated gradually under prolonged and moderate hypoxia. In addition, HIF-1α regulated anaerobic glycolysis and cell death, closely associated with GLUT1 and p53 pathways (36). Several studies about neuroblastoma that was also prevalent in children have also identified an association between high HIF-1α and low tumor stages, as well as better prognosis (37), which supported the potential protective effect of the high expression of HIF-1α in ACP patients.

Currently, little is known about the immune microenvironment in ACP, limiting the further use of targeted therapies in clinical practice. Thus, we comprehensively analyzed the immune infiltration of ACP. Our research found that ICI levels in ACP were positively correlated with HIF-1α, which proved that patients with high HIF-1α expression had more active immune responses, which was consistent with the HIF-1α-counteracted immunosuppression reported by Sonja (38). Hence, a high expression of HIF-1α and milder clinical symptoms in ACP patients may be achieved by increasing the level of adaptive immune response. Multiple studies have also detected a high level of inflammatory molecules in the cystic structure of ACPs including interleukin families, chemokines, and immune checkpoints, indicating that the immune checkpoint blockade (ICB) therapy might be a promising therapy for ACP patients (6, 39). We also analyzed the expression of immune checkpoints; PD-1/PD-L1 was positively correlated with HIF-1α, especially PD-1, and the correlation coefficient with HIF-1α reached 0.81. As an important protein involved in inhibiting immune signaling, PD-1/PD-L1 was an important regulator of immune activation (40), which suggests that patients with high expression might be sensitive to immune-targeted therapy. In addition, CTLA4, LAG3, and other chemokines were also well correlated with HIF-1α, providing help for the selection of subsequent treatment (41).

However, there are still some limitations in this study. On one hand, due to the lack of sufficient datasets of ACP, our sample size is still relatively small, which causes several bias errors. Hence, some results are still needed to be repeatedly explored and validated in other associated studies. Moreover, due to the limitation of laboratorial technologies, there is still the lack of mature cell lines or animal models for ACPs up to date and we could not further perform cellular or animal experiments to further investigate the concrete role of HIF-1α in the mechanism of ACP. Finally, although we have supplemented necessary sequencing and immunofluorescence assays to validate the relationship between HIF-1α and immune cell infiltration, the potential role of HIF-1α in immune infiltration or immunotherapy for ACP patients remains to be explored in depth by *in vivo* and *in vitro* experiments.

## Conclusion

In conclusion, this study first proposed the potential correlation among HIF-1α, special clinical characteristics, and the process of growth and development in ACP patients. Immune microenvironment analysis further indicated that HIF-1α was significantly associated with immune activation and might serve as a potential therapeutical target for the immunotherapy in ACP. Integrated transcriptomic data help us to reconsider the novel potential role of HIF-1α in the pathogenesis and therapeutic targets for ACP, especially for child-ACP patients, providing a novel promising idea for the precision medicine of ACP.

## Data availability statement

The original contributions presented in the study are publicly available. This data can be found here: https://www.ncbi.nlm.nih.gov/bioproject/PRJNA870049/.

## Ethics statement

This study was approved by the Ethics Committee of the First Affiliated Hospital of Zhengzhou University (2021-KY-0156-002), and informed consent was written by all participants for their enrollments. Written informed consent to participate in this study was provided by the participants’ legal guardian/next of kin. Written informed consent was obtained from the individual(s), and minor(s)’ legal guardian/next of kin, for the publication of any potentially identifiable images or data included in this article.

## Author contributions

QG and JL contributed to data acquisition, analysis, figure presentation, and the drafting of the manuscript. JP and LZ participated in the process of data acquisition and immunofluorescence experiments. DS and MZ contributed to sample collection and data analysis. DX and FG contributed to figures presentation, revision of the manuscript and the design of the study. All authors contributed to the article and approved the submitted version.

## Funding

This work was supported by grants from China Science and Technology Exchange Center (2021YFE0204700).

## Acknowledgments

We would like to thank all patients participating in this study.

## Conflict of interest

The authors declare that the research was conducted in the absence of any commercial or financial relationships that could be construed as a potential conflict of interest.

## Publisher’s note

All claims expressed in this article are solely those of the authors and do not necessarily represent those of their affiliated organizations, or those of the publisher, the editors and the reviewers. Any product that may be evaluated in this article, or claim that may be made by its manufacturer, is not guaranteed or endorsed by the publisher.
