# Supplementary figures and images for: Integrative analyses identify HIF-1α as a potential protective role with immune cell infiltration in adamantinomatous craniopharyngioma
Source: Front Immunol. 2022 Aug 24;13:949509. doi: 10.3389/fimmu.2022.949509 (PMC9450013; doi:10.3389/fimmu.2022.949509)

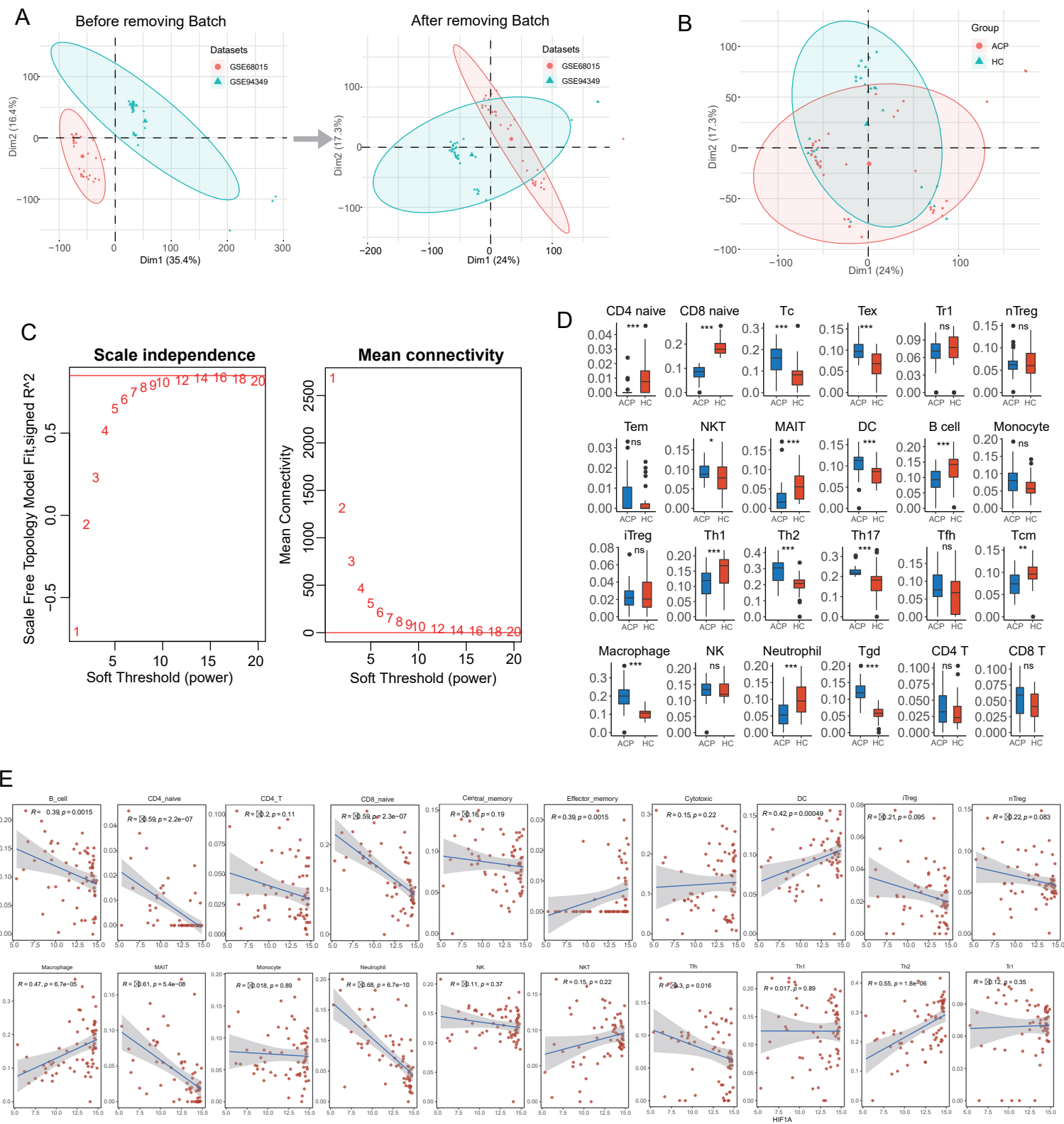

Supplement: Supplementary file 1 [file DataSheet_1.pdf]

A

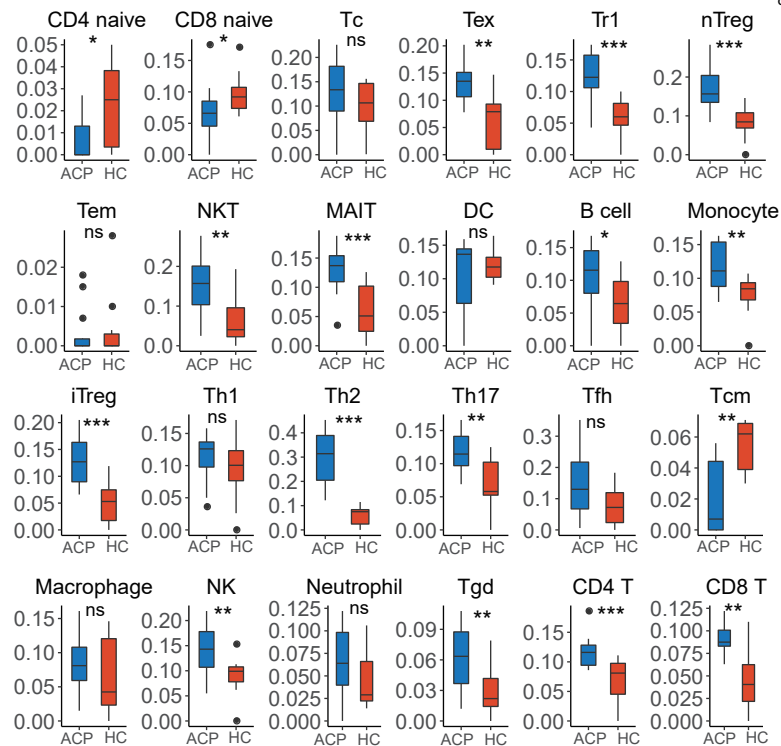

B

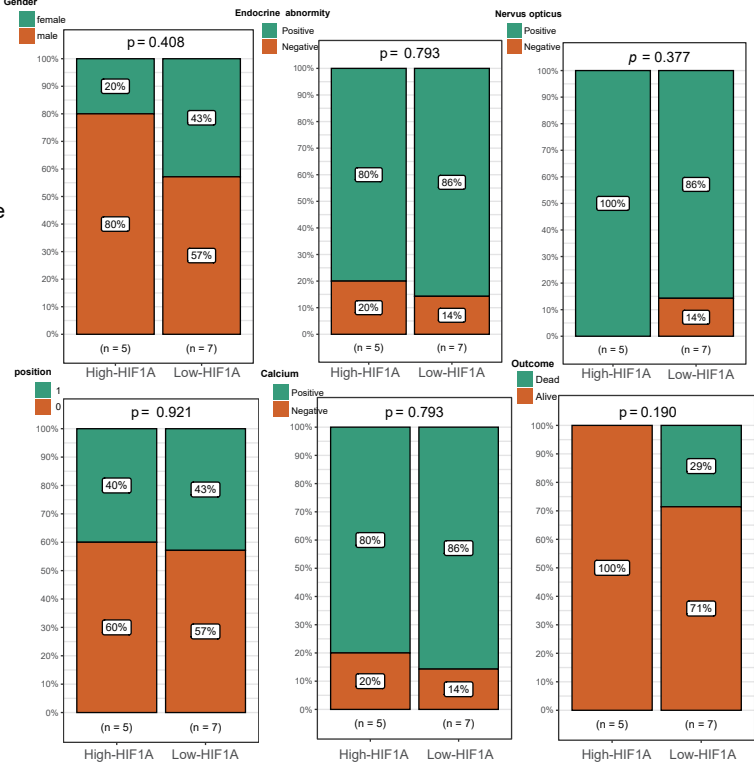

C

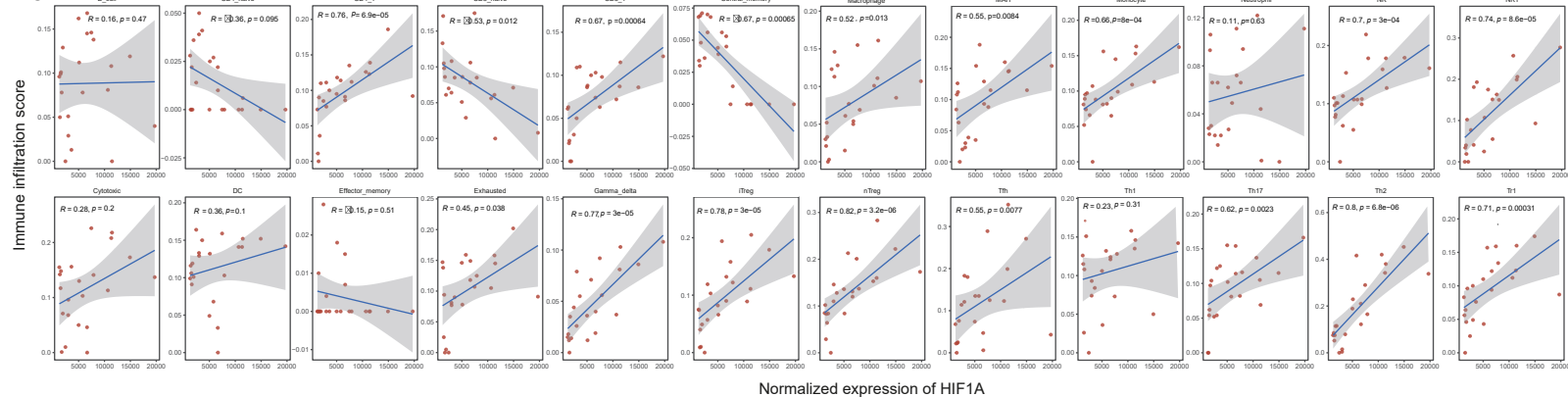

Supplement: Supplementary file 2 [file DataSheet_2.pdf]
